# Experiencing Retirement Through Teaching: Productive Engagement in Later Life across Two Cultures

**DOI:** 10.1007/s10823-026-09560-5

**Published:** 2026-01-28

**Authors:** Giuliana Casanova, Joyce Weil, Margarida Cerqueira

**Affiliations:** 1 Department of Education and Psychology, University of Aveiro, Aveiro, Portugal; 2 School of Medicine and Biomedical Sciences, University of Porto, Porto, Portugal; 3 Department of Health Sciences, Towson University, Towson, MD USA; 4 School of Health Sciences, University of Aveiro, Aveiro, Portugal

**Keywords:** Older adults, Peer-educators, Lifelong learning, Productive engagement, Cross-cultural comparison, Well-being

## Abstract

As life expectancy rises globally, many older adults seek meaningful opportunities to remain active after retirement. One such role is that of the peer-educator, in which older adults contribute their knowledge and experience by teaching age-based peers within lifelong learning organizations. This qualitative, cross-cultural case study explores the experiences of 34 older adult peer-educators aged 61 to 84 in Porto (Portugal) and Florida (U.S.). Guided by the cross-cultural productive engagement model and using reflexive thematic analysis of semi-structured interviews, this study explored motivations for participation, perceived rewards and challenges, organizational factors shaping engagement and perceived impacts on well-being. Participants were predominantly highly educated, with many reporting prior professional or teaching experience. Across both contexts, peer-educators described sharing knowledge, building community and reciprocity as central motivators and rewards, while designing meaningful and inclusive classes as a common challenge. Suggested organizational improvements focused on infrastructure and outreach; alongside context-specific needs. Overall, findings position the peer-educator role as a form of productive engagement in later life, demonstrating how contributing through teaching can support psychological well-being, mental stimulation, and sustained social participation, while underscoring the importance of organizational and cultural contexts in shaping meaningful opportunities for continued engagement.

## Introduction

Increases in life expectancy and health span are transforming how older adults experience later life. Older adults are often more educated, retire with accumulated skills, and seek continued engagement in society (Sibai & Hachem, 2021). Globally, the 65 years of age and older population is projected to triple by 2050 (UNESCO, 2022), creating both challenges and opportunities that require recognition of older adults’ contributions and new pathways for active engagement (Sibai & Hachem, 2021). Lifelong learning is one key avenue for promoting well-being, inclusion, and purpose in later life (Narushima et al., 2018). While typically positioned as learners, many older adults also serve as peer-educators, teaching age-based peers and fostering solidarity, mutual learning, and community cohesion (Choi, 2007). Yet research on these peer-educator roles, particularly in cross-cultural contexts remains limited.

## Theoretical Background

Engagement as peer-educators can be traced to early theories, such as Activity Theory (Havighurst et al., 1961), which links well-being in later life to continued participation in meaningful roles (Diggs, 2008; Daniels et al., 2010). This theory, however, has been critiqued for its narrow interpretation of what is valued in later life and it has limited attention to heterogeneity in later life, particularly differences related to class, gender, race and broader societal inequalities (Katz, 2000). The concept of productive aging has placed value on societal and individual benefits through paid or unpaid roles such as teaching, volunteering, caregiving (Butler & Gleason, 1985; Butler et al., 1990; Morrow-Howell et al., 2001; Sherraden, 2001; Schulte et al., 2018; Cole & Macdonald, 2020; Su, 2025). Within this framework, productive engagement refers to activities through which contributions occur, underscoring both individual and collective gains (Morrow-Howell & Greenfield, 2010; Morrow-Howell & Mui, 2013; Matz-Costa et al., 2014; Gonzales et al., 2015; Yen & Lin, 2018; Sia et al., 2021; Casanova, 2022; Pinazo-Hernandis et al., 2022; Xie & Han, 2024). Morrow-Howell and Wang’s Cross-Cultural Framework on Productive Engagement in Later Life allows for or both culture specific and cross-cultural research combining earlier WHO’s and Bass and Caro’ productive engagement model. Their model expands earlier perspectives by highlighting how participation is shaped by individual motivations and sociocultural contexts (Morrow-Howell & Wang, 2012). This study is conceptually grounded in this framework which provides a structural lens to examine how individual, organizational and societal factors shape older adult´s opportunities to engage in meaningful activities across diverse cultural contexts, it categorizes activities into social and leisure pursuits, self-care, capacity building, and economically valuable roles. Their model highlights how engagement is shaped by three key dimensions: antecedents, organizational capacity, and outcomes.

Antecedents refer to the individual and structural characteristics that shape opportunities for engagement. In this study, antecedents were captured through the sociodemographic characteristics of peer-educators, including age, gender, living situations, prior teaching experience, educational attainment, volunteer vs. paid status.

Organizational capacity is examined through peer-educator´s descriptions of their teaching environments and their suggestions for organizational improvements, reflecting how organizational support and constraints are experienced at the participant level.

The outcomes are considered at three levels: at the individual level, the analysis focuses on how peer-educator themselves experience their teaching roles, including their motivations, perceived rewards, challenges, and the impact on their overall well-being. At the organizational level, through educator´s reflections on how their participation sustains the functioning of lifelong learning organizations. Finally, at the societal level, outcomes are interpreted through the broader lens of how older adults’ contributions as educators generate social capital, and challenge ageist assumptions.

While criticized for privileging economic contributions (Dommaraju & Wong, 2021); Mathews, (2023), studies often link productive activity with self-reported well-being and purpose in later life (Baker et al., 2005; Gonzales et al., 2015; Russell et al., 2019; Cole & Macdonald, 2020; Xie & Han, 2024). The very notion of “productivity” has been contested for potentially reinforcing exclusion, as it may privilege those who are able to remain active while marginalizing others (Estes & Mahakian, 2001; Morrow-Howell & Gonzales, 2023). Similarly, labels such as successful aging have been critiqued for setting narrow standards that can contribute to age-based discrimination. The term productivity has to be understood as a choice, an opportunity for older adults to engage in activities they find meaningful instead of an expectation to stay active and productive as an obligation (Estes & Mahakian, 2001; Morrow-Howell et al., 2001; Gonzales et al., 2015). Few studies have examined older adult´s experiences as educators across different cultural contexts (Brown, 1981; Kaye, 1982; Simson et al., 2002; Brady et al., 2003; Choi, 2007, 2009).

### Lifelong Learning Institutes (LLIs) and Universities of the Third Age (U3As)

Lifelong learning is the opportunity to continue to engage and have access to education throughout the lifetime. The European Commission 2018 and the United Nations 2030 Agenda for sustainable development aim at ensuring that lifelong learning opportunities are available for all ages (Blanco, 2025; Wu & Jiang, 2025). Participation in lifelong learning enhances well-being across psychological, cognitive, and social dimensions, yet little is known about how teaching affects in older adults themselves (Williamson, 1997; Merriam & Kee, 2014; Mackowicz & Wnek-Gozdek, 2015; Talmage et al., 2015; McWilliams & Barrett, 2018; Narushima et al., 2018; UNESCO, 2022; Antunes & Maia, 2023; Shi & Jiang, 2024; Wenzel et al., 2024).

The University of the Third Age (U3A) movement emerged in the early 1970 s as a pioneering model of non-formal education tailored to older adults. Initially launched in France through university-affiliated programs, U3As aimed to promote active engagement and lifelong learning in public university settings (Swindell, 1993; Formosa, 2011, 2014; Swindell et al., 2011; Midwinter, 2014). In contrast the British model, launched in 1981, adopted a self-help model, peer-led approach in which older adults acted as both learners and volunteer educators. These two functional models shape the philosophy and practice of U3As worldwide, with adaptations reflecting local, cultural, economic and organizational contexts (Huang, 2005; Formosa, 2014, 2019; Jun, 2014; Rynkowska, 2020; Casanova & Cerqueira, 2022). In the U.S, programs such as the Institute for Retired Professionals and Elderhostel paved the way for the Bernard Osher Foundation, now the largest provider of lifelong learning (Shinagel, 2012; Hansen et al., 2016; Talmage et al., 2018, 2019). Meanwhile, in Portugal, the first *Universidade da Terceira Idade* was established near the 1980s, with only 15 such organizations operating in 2001(Veloso, 2007; Pocinho, 2015). A significant expansion occurred after 2012, and by 2015, the number of “universidades senior” had risen to 230 (Pocinho, 2015). According to RUTIS, the current network of Universidades Sénior in Portugal, there are now 370 organizations serving 65,000 students and 7,500 educators (Silva, 2025). These organizations are often affiliated with municipalities, universities, or local associations and aim to promote active aging and social participation by providing educational, cultural, and social activities tailored to older adults (Veloso, 2007; Porcarelli, 2019; Casanova et al., 2022).

#### Productive Engagement: Older Adults in Educator Roles

Although lifelong learning initiatives have primarily emphasized older adults as students, research also highlights the contributions of those serving as educators or facilitators (Simson et al., 2002; Choi, 2007). Peer-educators in these settings are typically not required to hold formal teaching credentials, but rather are selected or self-select based on their willingness and ability to share knowledge, skills, or interests with their contemporaries in a collaborative, non-formal educational environment. Unlike traditional academic contexts, these environments prioritize collaboration, instead of formal grading systems and certifications or diploma attainment (Choi, 2007, 2009). Throughout this manuscript, the term “peer-educator” refers specifically to retired older adults teaching or facilitating learning among their peers, rather than to professionally trained educators, or to interventions evaluated for pedagogical efficacy.

This research aims to profile older adults in Florida, U.S., and Porto, Portugal (demographics and backgrounds), explore motivations for engagement, rewards and challenges in their roles, recommendations to improve organizational practices and assess impact on post-retirement well-being.

## Methodology

### Study Design

This study employed a qualitative, cross-cultural case study design (Bartlett & Vavrus, 2017; Yin, 2018; Karlin, 2019; Pauluzzo, 2020) to examine the experiences of older adult peer-educators in lifelong learning organizations in Florida, U.S. and Porto, PT. The study was conceptually grounded in the cross-cultural productive engagement framework (Morrow-Howell & Wang, 2012), which informed interpretations of antecedents, organizational capacity and outcomes, while data were analyzed using reflexive thematic analysis (Braun & Clarke, 2016, 2021a, 2023; Trainor & Bundon, 2021; William, 2024), selected for its interpretivist foundations and recognition of themes as actively constructed through engagement with the data.

#### Study Setting, Participants, and Recruitment

Participants were older adult peer-educators in Lifelong Learning Institutes (LLIs) in Florida, U.S., and Universities of the Third Age (U3As) in Porto, Portugal. Both regions have experienced an increase in their older adult populations with a similar 20% rise (DOEA, 2021); WHO, 2023). Inclusion criteria were: age 60 or older, retired from full-time work, a citizen of the study country, and currently teaching in a lifelong learning organization for older adults. A total of 34 participants were recruited (17 per country).

#### Data Collection

This study was approved by the 2022/CE/P14(P392/CETIS/ICBAS;Institute of Biomedical Science Abel Salazar of the University of Porto; ICBAS-UP Ethics Board. Recruitment began in June 2022 in Florida and March 2023 in Porto, via email invitations to lifelong learning organizations. Administrators served solely as the initial organizational point of contact and assisted by identifying potentially eligible peer-educators and distributing study information. Participation was entirely voluntary. Administrators either shared interested participant´s contact information with the researcher with the participant´s permission or facilitated interview scheduling based on participant´s stated availability. In cases where contact information was shared, participants were contacted directly by the researcher via email or phone to arrange the interview. To minimize potential coercion, administrators were not involved during any of the process nor were they informed of which educators agreed or declined to participate. All consent procedures were conducted individually by the lead author prior to participation. Written informed consent materials described the study purpose, procedures, confidentiality protections and participant´s right to withdraw at any time without penalty. No incentives were provided.

A purposive sampling strategy was used to recruit participants. Sampling decisions prioritized depth of experience and diversity of perspectives rather than saturation (Braun & Clarke, 2021b). Semi-structured interviews were conducted in person at each participating organization. Interview locations varied depending on the physical spaces available within each organization and included classrooms, meeting rooms or other designated private areas provided by the organization. These settings were selected to ensure participant comfort and confidentiality. Some interviews were also conducted via Zoom, depending on the participant’s preference. All interviews were audio-recorded, transcribed verbatim and averaged approximately 30 min. Interview were conducted in English (U.S) and Portuguese (Portugal); Portuguese interviews were translated into English by a native speaker. Data were stored on password-protected devices accessible only to the lead author.

The interview guide elicited open-ended reflections on motivations, perceived rewards and challenges, organizational support, and impacts on well-being. For example, questions included “Can you tell me when you became a peer-educator at this organization and what motivated your decision?”; “Since becoming a peer-educator, what challenges and rewards have you experienced in this role?”; “are you also a student at this organization?”. The full guide is available upon request. All interviews were conducted by the lead author, whose gerontological training and trilingual fluency supported reflexivity and cross-cultural sensitivity. A brief sociodemographic questionnaire was administered as part of the interview to capture participant characteristics.

##### Data Analysis

Data were analyzed following Braun and Clarke’s (2021b) six-phase Reflexive Thematic Analysis (RTA) approach: familiarization, coding, theme development, review, definition and reporting. Combining both inductive (data-driven) and deductive (theory-informed) approaches. The first author led the analysis, beginning with data familiarization through repeated reading of transcripts and reflexive note-taking. Coding was conducted interactively in NVivo14, with codes generated to capture both sematic and latent meanings. Coding was iterative and recursive, with ongoing movement between data extracts, codes and patterns. Themes were actively constructed through reflexive engagement with the data. Consisted with RTA´s epistemological assumptions, the analysis did not involve intercoder reliability or consensus coding. Early discussions of a subset of transcripts with a supervisor functioned as reflexive dialogue to support analytic depth, not as a quality check or validation exercise. Themes were developed separately for each country to preserve contextual specificity and were subsequently examined comparatively to identify cross-cultural patterns and distinctions.

##### Trustworthiness

Analysis rigor was supported through the alignment with the principles of reflexive thematic analysis (Braun & Clarke, 2023). The study was located within an interpretative qualitative paradigm, with themes understood as meaning-based patterns constructed through the researcher´s engagement with the data. Methodological choices including purposive sampling, depth-oriented analysis and absence of saturation claims were theoretically aligned with reflexive TA. Reflexivity was integral to the analytic process and enacted through ongoing memo-writing and critical reflection (Braun & Clarke, 2016, 2021a, 2023).Theme development focused meaning-based themes rather than topic summaries. Rather than relying on member checking, quality was demonstrated through theoretical coherence, reflexive transparency, and the production of compelling, well-supported interpretative account.

## Results

### Sociodemographic Profile

#### United States Sample

The U.S. sample included 17 retired older adults, aged 61 to 84 years (Mean age: 73.8). A larger percentage of participants were women (58.8%, *n* = 10). Education attainment was high among U.S. peer-educators, 41.2% (*n* = 7) held a master´s degree, 29.4% (*n* = 5) held a doctorate, 23.5% (*n* = 4) held a bachelor´s degree, and one participant (5.9%) had completed technical school. Over half of the participants (52.9%, *n* = 9) lived with a spouse, while the remainder lived alone (47.1%, *n* = 8). Most participants reported prior teaching experiences: 82.4% (*N* = 14) with a mean of 11.4 years (SD = 7.6) of teaching. One average number of classes taught was 2.0 (SD = 1.2). Paid positions were common in the US group (41.2%, *n* = 7), while volunteerism was 58.8% (*n* = 10). In addition, 41.2% (*n* = 7) of participants attended classes as students. US peer-educators most commonly reported self-initiating their involvement (47.1%, *N* = 8), followed by invited by staff (29.4%, *N* = 5) or by peer/other educators (23.5%, *n* = 4).

#### Portuguese Sample

The Portuguese sample included 17 retired older adults, aged 65 to 83 years (Mean age: 72.5). Women comprised a larger percentage (64.7%, *n* = 11). Educational attainment was also high: 47.1% (*n* = 8) had a bachelor´s degree, 29.4% (*n* = 5) a master´s degree, 11.8% (*n* = 2) a doctorate and 11.8% (*n* = 2) had completed technical school. Most participants lived with a spouse (82.4%, *n* = 14), while the remainder lived alone (17.6%, *n* = 3). Nearly all had prior teaching experience (94.1% *n* = 16), with a mean of 9.3 years (SD = 4.5). On average, they taught 1.3 classes (SD = 0.5). Volunteerism was predominant (94.1%, *n* = 16), with only one participant holding a paid position (5.9%). Additionally, 41.2% (*n* = 7) also attended classes as students. Most participants were invited by staff (76.5%, *n* = 13), followed by being recruited by peers/other educators (5.9%, *n* = 1) or self-initiating their involvement (11.8%, *n* = 2). See Table 1 for sociodemographic characteristics of peer-educators in the Portuguese and the U.S. samples.

**Table 1:** Sociodemographic characteristics of Peer-Educators in the USA and Portugal samples

Category	Characteristic	USA (*n* = 17)	Portugal (*n* = 17)
Demographics	Age range (years)	61–84	65–83
	Mean age (years)	73.8	72.5
	Women (%)	58.8	64.7
	Men (%)	41.2	35.3
Living situation	With spouse (%)	52.9	82.4
	Alone (%)	47.1	17.6
Educational attainment	Bachelor’s degree (%)	23.5	47.1
	Master’s degree (%)	41.2	29.4
	Doctorate degree (%)	29.4	11.8
	Technical school (%)	5.9	11.8
Teaching background	Prior teaching experience – Yes (%)	82.4	94.1
	Prior teaching experience – No (%)	17.6	5.9
Volunteer status	Volunteer – Yes (%)	58.8	94.1
	Volunteer – No (%)	41.2	5.9
Participation	Attending as student – Yes (%)	41.2	41.2
	Attending as student – No (%)	58.8	58.8
Pathway to teaching role	Applied themselves (%)	47.1	11.8
	Invited by staff (%)	29.4	76.5
	Invited by friend (%)	11.8	5.9
	Invited by another educator (%)	11.8	5.9

### Thematic Analysis

Results are presented as overarching themes across both the United States and Portuguese samples, followed by country-specific themes that highlight unique themes. Illustrative quotes from participants are included to anchor the interpretation of each theme.

### Research Question 1: Reasons for Becoming an Older Adult Peer-Educator at this Organization

Across both samples, participants described overlapping motivations for becoming peer-educators, alongside motivations that were more context-specific.

#### Sharing Knowledge and Lived Experience

Across both contexts, peer-educators emphasized a desire to share accumulated knowledge from professional and life experiences.

Bob, U.S.A., 84 years old and a peer-educator for the past 12 years, explains: “*I really wanted at this time to share some of my knowledge*,* I understand I didn´t know what was the level of learning or study of other students and I had like an urge*,* to share some of my knowledge and it was received well by them*”. Lígia, PT, 71 years old, peer-educator for 7 years explained: *“And in fact*,* I thought it was worth coming here to give a little of what I did in my life*”.

#### Reciprocity and Co-learning in Later Life

Participants in both samples highlighted reciprocal learning; describing classrooms as spaces of mutual exchange rather than one-directional. Sandra, U.S.A, 79 years old and a peer-educator for 3 years, shared:

I think this is a give and take that this environment provides, from the educator´s point of view, as well as the student, they provide a great service I think, for people to continue to learn, everybody comes with a different set of baggage. And that contributes to the interesting part of the class, there is a give and take, there is a lot of conversation, that back and forth.

Edna, PT, a 71-year-old peer-educator for 4 years, shows the mutual and equal learning process:

I often feel more among peers, they are seniors, they are people more or less my age and therefore, I am not so much a teacher, but as someone equal to them, who can contribute or give something of their ability and their experience. I am in favor of the common enjoyment; we have a common attitude of sharing.

Motivations also differed by national context.

#### United States: Passion, Purpose, Flexible Commitment and Personal Enjoyment

U.S participants highlighted personal fulfillment and enjoyment as central motivators. Teaching provided meaning and continued growth in retirement. As Susan, 71 years old, 21 years as a peer educator explained: “*I really have to do this*,* it is my purpose*,* and I don´t want to do anything else*,* I mean I want to teach everybody young and old… It´s given me a new purpose*,* it´s really been one of the loves of my life*”. Participants also valued the low-pressure environment. Lisa, 78 years old, peer-educator for 5 years added the freedom from traditional academic settings:

It [at the Lifelong Learning Institute] was fewer hours and easier and I was happier… There is no homework, nobody´s under pressure, they are only under whatever pressure they impose on themselves, which is quite frankly a lot sometimes, but that is self-imposed.

Rather than viewing teaching as a burden or obligation, U.S. participants emphasized the personal enjoyment and emotional fulfillment derived from the role. Associating teaching with energy, joy and connection. Lisa, 78 years old and an educator for 5 years stated: “Is this happier for me? You bet! it is a much more pleasant experience, if it weren´t, they wouldn´t have been able to talk me into staying for the last year so yeah, it is fun”.

#### Portugal: Lifelong Vocation and Civic Duty

Portuguese participants framed teaching as a continuation of a lifelong vocation and a moral obligation to give back to society. Several had professional experience teaching younger generations and saw this role as a natural extension of their identity. This sense of vocational passion was linked to self-fulfillment. As explained Edna, a 71-year-old peer-educator for 4 years: “*It was very natural for me to integrate as a teacher*,* because it was what I have always done in life. It is what I have always liked to do and that fulfills me a lot”*, others described their participation in their organization as a moral obligation or service to others. As Edna, a 71-year-old peer-educator for 4 years, said: “*As a teacher I do it with a spirit of social contribution that for me is very gratifying*,* there is a kind of exercise of citizenship*”.

### Research Question 2: Rewards Experienced as a Result of Being a Peer-Educator at this Organization

Participants across both the U.S and Portuguese samples described several rewarding aspects of being a peer-educator, with some experiences shared across countries and others more specific to the U.S.

#### Building Community and Meaningful Connections

Social bonds and connections were rewarding in both U.S and Portuguese participants. Participants spoke of the importance of making friendships and meaningful bonds. Sandra, U.S.A., a 79-year-old peer-educator for 3 years said: *“I have met a lot of interesting people*,* my whole goal when I came here was to meet people”* Portuguese participants was the formation of meaningful bonds which in return created a sense of belonging. Cátia, PT, a 66-year-old peer-educator for 15 years, said: “*The rewards are great*,* the friendships I made and that I continue to make*,* the friends I have here are very good”.*

#### Student Development and Growth

Seeing student´s progress and develop new skills was rewarding in both samples. Tom, U.S.A., 67 years old, peer-educator for 4 years commented about the fulfillment he felt in helping artists grow:

What amazes me is how truly good potential artists are out here, that haven’t really exercised this, there are some very talented people out there, and there´s some people that didn´t even know they had talent, which is also amazing, people that said I never thought I could be an artist, the great reward in the world is seeing people that are learning a new skill, and the thought that they can’t learn at a certain age is BS, they are learning.

Patrícia, PT, a 68-year-old peer-educator for 1 year added that seeing student´s development is the real return: *“The reward is that we get to see that we have achieved something*,* maybe it wasn´t the original goal*,* but we get them to be more independent*,* to be able to work alone*”.

#### United States: Mutual Learning, Personal Growth and Self-enrichment

*U.S.A* educators expressed a reward was the exchanging of ideas, being exposed to new perspectives and continuing to grow through the interaction with their peers. For example, Lana, 78 years old with 23 years as a peer-educator reflected:

Because they are old people, I love it, because I think I am learning from them, every time I give a lecture of great interest, people are fascinated by the subject. They all come up and they tell me what happened to them, or what they thought, I love that that feeling of learning from other people. It´s one of the rewards of doing this.

Another reward several U.S. participants reflected on was how this activity contributed to their own personal development. Some described this as a “selfish” benefit, not out of egoism, but to express how personally fulfilling and intellectually stimulating this experience was for them. Patrick’s, a 73-year-old peer-educator for 4 years quote serves as an example: *“The rewards are the opportunity to create a class*,* putting together a presentation*,* doing the research*,* so it really keeps me inspired*”.

### Research Question 3: Challenges Encountered Working as an Educator at their Organizations

Participants described a range of challenges encountered in their role as peer-educators, with some shared across both countries and others specific to the context of their organization.

#### Designing Engaging, Meaningful and Inclusive Classes

Both U.S and Portuguese participants emphasized the effort required to create stimulating and relevant content for diverse learners. This included considering varying levels of prior knowledge, learning preferences and accessibility needs. Julie, U.S.A., who is 77 years old and a peer-educator for 4 years, also commented on the planning hurdle, explaining “*the issue of having all the research I have to do before we meet*,* because it is my responsibility to provide some of the background and stuff*,* and keeping people interested so they keep coming back”.* Cátia, PT, 66 years old, spoke about the challenge in designing classes for such different groups of learners: *“It was to try to mentally organize and structure the classes so that they understood it and were satisfied with what they heard”*.

#### Managing behavior and Age-Related Classroom Challenges

Both U.S. and Portuguese participants described challenges in managing classroom dynamics, including disruptive behavior, dominant personalities, and accommodating students with hearing, cognitive or mobility limitations. As well as adapting their teaching approaches to meet the diverse needs of older adults, such as reduced attention spans and sensory impairments. Mark, U.S.A., 73 years old, peer-educator for 7 years, said:

It is very interesting for me to learn that you have the same type of stuff going on here. Now there´s people that come in and fight over, oh my gosh, it´s almost like middle school drama. I didn´t expect that at all, I didn´t, because they are adults. I feel like I still have to deal with middle school drama.

Lígia, PT, who is 71 years old and a peer-educator for 7 years, said:

There are different levels of knowledge in the class itself, therefore, there are those who know something, there are those who know nothing and this is a challenge. It’s one of the hardest things being able to give an English class to someone who doesn´t know anything and having others who already know a lot more.

#### United States: Inadequate Physical Environment. U.S. Participants Voiced Frustration Over Logistical and Infrastructure Limitations

From poorly equipped classrooms, limited accessibility features, outdated technology among others. Narrative from two peer-educators with very different lengths of teaching reflect the same theme. Susan, who is 71 years old and a peer-educator for 21 years, stated: “The *challenge is I work in the theater lobby*,* and sometimes it´s very noisy*,* the space is not enclosed*,* another challenge is sometimes it´s cold”.* Dave, an 83-year-old peer-educator for 1 year also added: “*the challenge is the lighting system in the room they have for art*,* the room itself it’s a utility room for all kinds of activities*,* a universal room I would say*”.

#### Research Question 4. Suggestions for Improvements within the Lifelong Learning Organization in Order to Improve Older Adults’ Experiences

Participants across both countries provided insights on way to improve lifelong learning practices, with several common suggestions as well as context-specific recommendations.

#### Enhanced Physical Infrastructure and Technical Support

Many educators highlighted the need for better learning environments, including larger or more comfortable classrooms, updated technology and improved facilities. Susan, U.S.A reflected: *“more space*,* a classroom with a door*,* a closed space where you really hear very minimally outside”* Patrícia, PT, spoke about how this unmet need impacts her learners: They have to bring computers, because we don´t have computers for them, each of them brings their own computers, and they are very old ones. The university does not have computers for the students. If we had better ones, it would be easier for them to work.

#### Membership Growth, Participation, and Commitment

Both U.S and Portuguese participants noted the importance of attracting more students to enhance class dynamics, motivations, and course viability. For example, Julie, U.S.A., 77 years old and a peer-educator for 4 years, said:

The program needs more volunteers actually, and we should have more people. Even yes, I came from teaching that’s a given, but people who didn´t come from teaching, I mean people who are attorneys, judges, doctors, whatever else, they could contribute to the program, and I would like to see more classes being taught by peers, and I don´t know how we can go about doing that, about getting people to do it.

At the same time, others commented about the need for outreach for more student participation and enrollment. Cristiana’s, PT, narrative explains the situation:

Recruitment it´s always difficult, there are a lot of good people out here in this city who could be volunteering, passing on knowledge or giving of themselves, I don´t know how we could recruit these people.

#### United States: Improved Marketing and Community Outreach

U.S. educators identified a clear need for stronger promotional efforts, particularly in reaching older adults who may be unaware of the organization´s existence. Jennie, 61 years old and a peer-educator for 2 years, said:

You know I’ve had to cancel classes several times because I don´t have enough registration, but I know they had two big events to try to get more members and more people, and they´ve asked me to think about rebranding the class to we get more people interested, so I might need some help from them on that.

They emphasized the importance of reaching out through social media outlets, local partnerships, etc. to give more visibility to these types of organizations. Lisa, 78 years old and a peer-educator for 5 years, explained the need for more promotion:

I need at this point, we need marketing, we need to be somehow more, there needs to be a spotlight on the program, because it is a really good program, and I think a lot of people just don´t know about it, I always felt marketing and promotion are really important especially when your numbers are down.

#### Portuguese: Better Pedagogical Practices, Training for Older Adults

Several Portuguese participants brought knowledge or practical experience, but some felt unprepared to manage diverse classrooms or deliver a class in engaging, age-appropriate ways. Patrícia commented about her feeling of a lack of pedagogical training for her role:

Maybe the ideal would be to have a computer science teacher, a real computer science teacher, because there are several techniques for teaching, we don´t have those skills, what we know is what we learned here. Maybe we could reach the students better, if there was a teacher who was really trained.

Others brought up how teaching older adults differs from teaching younger learners. For example, Célia noted:

This is a totally different kind of teaching. It is good for us in terms of practicing, but at the pedagogical level? For example, how do we treat the students? Even managing a class. Sometimes we hear comments in the cafeteria like: “She knows how to be a teacher”, “He doesn´t know how to teach”. Therefore, a person can know very well how to play an instrument but it is necessary to know how to pass on that knowledge to the students.

#### Research Question 5: Impact on the Educator Role on Well-Being

In this final section, the peer educators reflected on what their role brought them, rather than focusing on what they bring to their learners in their own day to day roles. Both U.S and Portuguese educators reflected on its impact on their emotional, cognitive and physical well-being.

#### Emotional Fulfillment, and Social Connection

Peer- educators’ narratives stressed the impact engaging in this activity had on their emotional well-being. Educators described a sense of emotional nourishment that added structure and vitality to their lives post-retirement. Sandra, U.S.A., who is 79 years old and a peer-educator for 3 years, said: “*my life it is definitely been enhanced*,* it makes me feel much more animated and energized*”. They valued the opportunity to build lasting friendships and interpersonal bonds. António, PT, 78 years old and a peer-educator for 3 years, said: *“this is stimulating*,* because it makes me feel alive*,* active*,* and it makes me be curious*,* to seek to know more*,* and also try to improve the way of reaching people”*.

#### Cognitive Engagement and Mental Stimulation

Participants also emphasized the mental health benefits of participating in this activity. It helped them feel mentally sharp, challenged and curious. Peer educators mentioned that: *“I think my brain is working harder than it´s worked in a long time”* (Tom, U.S.A., 67, peer-educator for 4 years) and that “*for my own mental health is really a valuable asset*,* and I hope it would be for a lot of other educators”* (Earl). João, PT, commented: *“it forces me to read*,* to study*,* and so I learn automatically”*.

#### Physical Benefits

Some participants described how being involved in this activity especially if they taught classes such as physical education, movement or dance felt more energetic and physically engaged. A comment from Mike, U.S.A., illustrates the physical dimension: “*physically definitely*,* because I teach the class*,* and it´s two hours*,* and I am sweating just as much as they are”.* Celeste, PT, expanded upon this concept:

The impact of being a teacher, is doing the exercises, it forces me to exercise once a week. I had problems (heart surgery recently), but I like to exercise, and that obligation to come here and give class, I say “obligation” because it forces me to exercise. It makes a big difference if I didn´t come.

An overview of common and country specific themes in peer-educator experiences can be seen in Table 2.

**Table 2:** Overview of common and country-specific themes in peer-educator experiences

Research question	Common Themes	US-Specific Theme	PT-Specific Theme
1. Reasons for becoming peer educator	Sharing knowledge and life experience and reciprocal learning from students	Passion and personal purpose, personal enjoyment and flexible commitment	Lifelong vocation, teaching as a calling and civic duty
2. Reward of being an educator	Building community/social connections; witnessing student growth and development	Mutual learning, personal growth and self-enrichment	
3. Challenges encountered	Designing engaging, meaningful and inclusive classes Managing behavior and age-related challenges	Inadequate physical environment	
4. Suggested improvements to the organization	Better infrastructure and technical support Membership growth, participation and commitment	Improved marketing and community outreach	Better pedagogical practices and training
5. Impact on well-being	Emotional well-being (purpose, connection) Mental stimulation and creativity Physical impact		

#### Findings Beyond the Research Questions: Peer Educators’ Narratives about Trade-Offs and Strain

While most participants described teaching as beneficial, a few noted trade-offs. Some mentioned conflicts with personal freedom, emotional strain in managing classes or sacrificing other activities. As one Portuguese educator talked about teaching as a commitment that limits their time for other activities: *“I don´t like to fail when I commit to things*,* but I want to be free*,* I can´t anymore because I have to teach”*. This perspective although less common, highlighted that even fulfilling roles can involve personal sacrifices and challenges.

Although beyond the initial research questions, participants in both countries reflected about the broader societal impact of lifelong learning organizations. Mark, U.S.A., 73 years old and a peer-educator for 7 years, advocated for lifelong learning-lessons: *“We´d have a better society if more people shared what they´ve done in life*,* that´s a really cool thing”.* Portuguese educators echoed such reflection, highlighting the role of senior universities in promoting socializations and combating loneliness. Francisco, who is 77 years old and a peer-educator for 15 years spoke of social connectivity: *“[students] are busy*,* and socialize*,* in addition to classes*,* people talk to each other*,* exchange experiences*,* they make friends”.* These reflections underline the societal value of lifelong learning as both an educational and social resources in later life.

## Discussion

This study contributes to literature on productive engagement in later life by examining the experiences, motivations and perceived impact of older adult’s servings as peer-educators within lifelong learning organizations from their own words and points of view. Framed through the concept of productive aging (Butler & Gleason, 1985; Morrow-Howell et al., 2001) and guided by the cross-cultural Productive Engagement Model (Morrow-Howell & Wang (2012), this study interprets peer-educator´s experiences through the model´s three key dimensions: antecedents, organizational capacity and outcomes. In this study, the model provided a structural lens to examine how individual characteristics, perceived organizational supports, and the resulting outcomes shape opportunities for productive engagement in diverse cultural contexts, focusing on the act of being a peer-educator.

Antecedents were reflected in the sociodemographic profiles which reflected a female-majority sample with high levels of educational attainment, consistent with prior research linking education and gender to later-life learning (Formosa, 2014; McWilliams & Barrett, 2018; Wenzel et al., 2024). Many participants had prior professional or teaching background, reflecting prior research on peer-education and later life teaching roles (Brown, 1981; Brady et al., 2003; Choi, 2007; Simson et al., 2008); such experience appears to facilitate entry into educator roles and sustained engagement in later life. Viewed through an intersectional lens, gender and education status likely influenced access to peer-educator roles, shaping participation and sustained engagement across contexts. Recruitment pathways differed: U.S participants often self-initiated involvement, while in Portugal, the majority were recruited by staff members, reflecting cultural and organizational norms. Portuguese participants more commonly volunteered, while paid roles were more frequent in the U.S., however, commitment to remain engaged was strong in both groups; aligning with research linking reciprocity and mutual benefit to sustained volunteerism (Morrow-Howell et al., 2014; Leung, 2016; Sung et al., 2023; Han & Zhang, 2025; He et al., 2025).

Organizational capacity was examined through participant´s suggestions for improvements. U.S peer-educators called for infrastructure, resources and outreach, whereas Portuguese participants highlighted strengthening pedagogical skills for teaching older learners. Consistent with the cross-cultural framework of Morrow-Howell and Wang (2012) these findings illustrate how perceived organizational supports and resources can either expand or constrain opportunities for productive engagement (Morrow-Howell et al., 2014; Sellon, 2014; Carr et al., 2015; Gonzales et al., 2015; Bárrios et al., 2018).

Finally, outcomes, were explored across both settings, participants emphasized fulfillment, purpose, and social connection, supporting prior work linking productive roles to meaning and reduced isolation (Simson et al., 2002; Strom & Strom, 2020; Su, 2025). Reflecting on previous research on peer-education in later life (Brady et al., 2003; Lightfoot & Brady, 2005; Choi, 2009), this study found that reciprocity and mutual learning were key rewards experienced by the participants, highlighting the value of shared learning in adult engagement. However, cultural nuances emerged: U.S participants emphasized individual enrichment, while Portuguese stressed collective and relational benefits, reflecting broader cultural orientations. These differences reflect broader cultural orientations and illustrate how productive engagement is shaped by national and organizational contexts.

Peer-educator roles also enhanced emotional, mental and physical well-being, paralleling volunteerism research (Morrow-Howell et al., 2014). Beyond individual outcomes, participants in both samples, stressed the value of lifelong learning organizations in promoting purpose, social bond, and smoother transitions into retirement (Morrow-Howell et al., 2014; Mackowicz & Wnek-Gozdek, 2015; Talmage et al., 2015); these findings underscore how educator roles can support identity continuity and post-retirement meaning. Such reflections challenge ageist narratives by highlighting how retirement does not mark the end of contribution or personal growth, instead it acts as a way of sharing accumulated life experience offering opportunities for older adults to thrive (Romaioli & Contarello, 2021). Peer-educators’ comments point to the need to address internalized ageism, which can limit perceived opportunities for engagement (Morrow-Howell, 2023, 2024). Ultimately, older adult peer-educators are not merely transmitting knowledge but cultivating community, modeling purposeful aging, and shaping their own post-retirement identities.

## Limitations

This study offers important insights into the experiences of retired older adults serving as peer-educators in lifelong learning settings across Florida, U.S., and Porto, Portugal and several limitations should be noted. First, as with most qualitative studies, purposively sample drawn from specific organizations may limit transferability to other setting or populations (Andrade, 2021). Data were collected at a single point in time restricting how motivations, experiences and commitment may change over time. Second, the sample consisted largely of highly educated participants, many of whom had prior professional or teaching experience. This may accurately reflect the experiences of individuals who tend to participate and lead lifelong learning initiatives, particularly in urban or university-affiliated contexts with the socioeconomic characteristics of the regions in which these organizations operate. As such, the findings may underrepresent the experiences of older adults from lower socioeconomic backgrounds or those with limited access to formal education.

Given the study´s cross-cultural focus, the design required translation of Portuguese interviews into English, and although care was taken, some cultural nuances may not have been fully captured. Women represented a modest majority of participants in both samples, which may have shaped the themes identified given possible gendered differences. Finally, the sample was limited to two urban settings, limiting applicability to rural or less-resourced contexts. Despite these constraints, the findings offer valuable cross-cultural experiences can inform lifelong learning organizations with similar goals.

### Implications for Policy, Practice and Research

Older adult as peer-educators should be recognize as active agents of productive engagement, fostering community, modeling purposeful later life and challenging ageists narratives. Policy frameworks about active and productive aging ought to include this activity alongside volunteering, caregiving, or part-time employment, ensuring visibility and resources at local and national levels. Cross-cultural perspectives, as shown here, reveal how different contexts shape meaning and practice, allowing countries to learn from each other (Morrow-Howell & Wang, 2012). Morrow-Howell and Wang’s Cross-Cultural Framework on Productive Engagement in Later Life allows for or both culture specific and cross-cultural research combining earlier WHO’s and Bass and Caro’ productive engagement model.

This study’s findings align closely with the World Health Organization´s Decade of Healthy Aging (2021–2030), particularly its focus on meaningful participation and social inclusion; and environments that support the abilities of older adults. They also support the United Nations 2030 Agenda, including goal 8 (promoting inclusive and productive activities) and Goal 4 (quality education and lifelong learning) (United Nations 2024; World Health Organization 2021). Peer-educator roles provide a practical mechanism for advancing healthy aging. At the municipal, regional and national levels, governments can promote visibility of these roles, allocate resources to sustain them, and support marketing and outreach strategies to expand participation. At the organizational level, targeted pedagogical training for older adults, investments in accessible infrastructure and technology can strengthen engagement.

At the organizational level, the results highlight the need for investment in accessible infrastructure, technological support and targeted pedagogical training tailored to teaching older learners. Such supports can enhance both educator effectiveness and student engagement, particularly in diverse classrooms.

From an equity perspective, the highly educated composition of the sample raises important questions about access to lifelong learning and teaching opportunities among older adults from lower socioeconomic backgrounds. Limited access to education, transportation, technology and organizational outreach may constrain participation for these groups, with implication for social inclusion. Future policy and practice efforts should consider ensuring that opportunities to teach and learn later in life are accessible, affordable and responsive to diverse socioeconomic realities.

Future research should extend beyond the U.S and Portugal, include rural and diverse contexts, and adopt longitudinal designs to capture sustained impact of engagement on well-being and identity. Additionally, building on these qualitative findings, future studies can explore deeper peer-educator programs using quantitative measures such as indicators of psychological well-being, life satisfaction and social engagement. Combining qualitative and quantitative approaches would allow for a more comprehensive assessment of engagement in later life and inform policy and practice. Recognizing older adults as both beneficiaries and drivers of lifelong learning aligns with global frameworks, ensuring opportunities for engagement are accessible, interest-driven and inclusive (Morrow-Howell & Wang, 2012; Mathews, 2023).

## Conclusion

This study shows while cultural nuances shape how older adults perceive and pursue peer-educator roles, the core impact of this activity has commonalities across borders. U.S participants preferred a more individualistic orientation, emphasizing personal growth and self-fulfillment, whereas Portuguese participants provided on a more collectivistic ethos, prioritizing civic duty, shared experiences and community bonds. Despite these differences, both groups agreed that teaching in later life foster’s purpose, strengthens social ties, and provides meaningful way to share knowledge and life experience.

The findings affirm that older adults in this study did not see themselves as passive recipients of service but active contributors to social capital, sustaining the vitality of lifelong learning organizations. Whether their contributions are voluntary or remunerated, the gains ripple outwards, from personal well-being, to student enrichment, to the strengthening of community life. Recognizing and supporting these roles is not only a matter of valuing older adults, but also investing in resilient, inclusive and age-integrated societies. Although the concept of productive aging has been critiqued for potentially excluding some older adults and overemphasizing economic value, our findings show older adult peer-educators view productive engagement as a choice, one that, when available, can enhance lives and empower older adults. Finally, this study illustrated that choosing to become a peer-educator in later life is not simply about knowledge transmission, but about connection, and meaning-making. In both the U.S and Portugal, older adults’ educators are using their agency, they are shaping lives, including their own.

## Data Availability

The datasets generated and or analyzed during the current study are not publicly available due to privacy and ethical restrictions but are available from the corresponding author on reasonable request.
